# Transfer of manualized CBT for social phobia into clinical practice (SOPHO-PRAX): a study protocol for a cluster-randomized controlled trial

**DOI:** 10.1186/1745-6215-13-70

**Published:** 2012-05-30

**Authors:** Stephen Crawcour, Eric Leibing, Denise Ginzburg, Ulrich Stangier, Jörg Wiltink, Jürgen Hoyer

**Affiliations:** 1 Clinical Psychology and Psychotherapy, Technische Universität/University of Technology Dresden, Institutsambulanz und Tagesklinik IAP-TUD GmbH, Hohe Straße 53, 01187, Dresden, Germany; 2 Abteilung Psychosomatische Medizin und Psychotherapie Zentrum Psychosoziale Medizin, Georg-August University, von-Siebold-Str, 5, 37075, Göttingen, Germany; 3 Institut für Psychologie - Klinische Psychologie und Psychotherapie, Goethe University Frankfurt, Varrentrappstr. 40-42, 60486, Frankfurt am Main, Germany; 4 Clinic of Psychosomatic Medicine and Psychotherapy, University Medical Center of the Johannes Gutenberg University of Mainz, Untere Zahlbacher Straße 8, 55131, Mainz, Germany

**Keywords:** Social phobia, Cognitive behavioral therapy, Remission, Phase-IV research, Training, Dissemination, Treatment, Outcome

## Abstract

**Background:**

Cognitive-behavioral therapy (CBT) is generally known to be efficacious in the treatment of social phobia when applied in RCTs, namely when the treatment manual is based on the Clark-Wells approach. However, little is known about the efficacy of manualized treatments in routine clinical practice (Phase IV of psychotherapy research). The present study (SOPHO-PRAX) is a continuation of a large multicenter randomized clinical trial (SOPHO-NET) and analyzes the extent to which additional training practitioners in manualized procedures enhances treatment effect.

**Methods/design:**

Thirty-six private practitioners will be included in three treatment centers and randomly designated to either training in manualized CBT or no specific training. The treatment effects of the therapies conducted by both groups of therapists will be compared. A total of 162 patients (*n* = 116 completers; *n* = 58 per condition) will be enrolled. Liebowitz Social Anxiety Scale (LSAS) will serve as primary outcome measure. Remission from social phobia is defined as LSAS total ≤30 points. Data will be collected at treatment begin, after 8, 15, and 25 sessions (50 min each), at treatment completion, as well at 6 and 12 months post-treatment.

**Discussion:**

The present CBT trial combines elements of randomized controlled trials and naturalistic studies in an innovative way. It will directly inform about the incremental effects of procedures established in a controlled trial into clinical practice. Study results are relevant to healthcare decisions and policy. They may serve to improve quality of treatment, and shorten the time frame between the development and widespread dissemination of effective methods, thereby reducing health cost expenditure.

The results of this study will not only inform about the degree to which the new methods lead to an improvement of treatment course and outcome, but also about whether the effects of routine psychotherapeutic treatment are comparable to those of the controlled, strictly manualized treatments of the SOPHO-NET study.

**Trial registration:**

ClinicalTrials.gov identifier: NCT01388231. This study was funded by the German Federal Ministry of Education and Research (SOPHO-NET: BMBF 01GV0607; SOPHO-PRAX: BMBF 01GV1001).

## Background

Social phobia (SP), also known as social anxiety disorder (SAD), represents a chronic and debilitating mental disorder characterized by persistent fears of one or more social situations in which the person is exposed to unfamiliar persons and expects to be scrutinized by others. The person affected fears acting (or showing anxiety symptoms) in a way that will cause embarrassment and humiliation [1]. DSM-IV-TR criteria indicate that, though such fears are recognized as unreasonable and excessive, exposure to such feared situations may invariably trigger anxiety, which may escalate to a situation-bound or predisposed panic attack. The fears in social phobia can cause clinically significant distress or impairment in social, occupational, or other important areas of functioning [1]. Epidemiological studies have also shown co-morbid psychopathology (other anxiety disorder, mood disorders, substance abuse) to be common for this disorder (for example [2]).

Prevalence rates have been found to vary across countries. A recent study by Ruscio *et al.*[3] found estimates of lifetime and 12-month prevalence at 12.1% and 7.1%, respectively. In Europe, a lifetime prevalence of 6.65%, and a 12-month prevalence estimate of 2.0% were found [4,5]. The age of onset tends to be located between preadolescence and early adulthood, for example between 10 and 16.6 years [6]. Onset at an age later than 25 years appears to occur less frequently [3,7].

Epidemiological studies have shown SP to be typically chronic in its course (for example [8]). Full remission from SP symptoms, though infrequent, tends to occur within 8 years when no treatment is provided [9,10]. Probably due to the overlap in the symptoms of shyness and SP (that is, heightened autonomic arousal in social situations (for example increased heart rate, blushing, sweating, [11], social skills deficits, such as low eye contact)), avoidance of social interactions, and cognitions reflecting fear of negative evaluation [12-14], those affected by the latter may not be easily detected and thus remain untreated [6].

Development and dissemination of efficacious treatments for SP is therefore in clear demand. CBT for social phobia is generally efficacious (for example [15,16]). RCT trials favoring the Clark-Wells approach [17] show the highest effect sizes [18,19], even outperforming standard pharmacological treatment [18] and suggesting specifically this approach to be distributed to routine clinical practice. However, to date a large disconnect between research and practice appears to hinder the development of psychology, both as a science and as a profession. Access to evidence-based psychological treatments has become a growing problem in the last 10 years. In spite of the support by healthcare authorities, the urgently needed evidence and consensus regarding the success of implementation of such treatments, in addition to the measurement of successful training outcomes, continues to be missing [20]. Specifically for social phobia, the quality and effectiveness of CBT in routine practice remains unknown.

To the best of the authors’ knowledge, there is also no current research: (1) directly examining the effects of additional training in a manualized and highly effective procedure on outcome in routine CBT for social phobia; and (2) testing the effectiveness of CBT for social phobia (in the control group), usually delivered in the private practices in Germany.

Hence, as a continuation of the Social Phobia Psychotherapy Research Network (SOPHO-NET, [21]), the present study (SOPHO-PRAX) is designed as a multicenter randomized clinical trial based on a standard protocol and a set of standardized measures for the assessment and treatment of SP. The present study protocol aims at testing the potential of the Clark and Wells CBT approach to the treatment of SP evaluated in the main trial of this research cooperation [21] in order to improve the effects of routine psychotherapies. It therefore appears important to investigate in detail to what extent such treatment methods can be transferred from controlled trials to the less structured setting of routine clinical care, and whether the healthcare system benefits from such developments. The rationale of this study thus represents Phase IV of psychotherapy research.

## Methods/design

### Study centers

The present study is being conducted by three trial sites, namely the Universities of Dresden (Prof. Dr. Hoyer), Frankfurt (Prof. Dr. Stangier), and Göttingen (Prof. Dr. Leibing), each coordinating at least 12 private practices.

### Participants

Patient inclusion and exclusion criteria are listed in Table 1. The sample fulfilling these criteria are considered representative for social phobia patients qualifying for treatment in an outpatient setting. Assuming a drop-out rate of 25% over a period of 2 years, a total of 162 patients are planned to be enrolled in the study (that is 54 patients in each center; see sample size calculation). Patients will only be able to enrol once in the present trial. Participants will be able to directly contact the trial centre in response to advertisements via the internet, flyers, or newspaper articles. Alternatively, they will be referred to by healthcare practitioners (that is, medical doctors or private practitioners in psychotherapy). Once the diagnostician confirms the patient’s eligibility for the study, the patient will select and contact a therapist from a list of candidates for treatment. Only private practitioners officially listed in the German chambers of psychotherapists will be recruited. Informed consent from both the therapist and the patient will be collected. All therapists will have to be certified in CBT. Inclusion criteria for therapists are broad (that is, any age and gender, living in the area of the trial center) as a function of the naturalistic aspects of the present trial. However, practitioners having recent training (within the last 5 years) in the Clark and Wells treatment of SP [17] will be excluded from the study. Measures of allegiance to the treatment condition and professional experience will be collected via self-report of the therapists as well as via expert ratings of video-documented sessions.

**Table 1:** Inclusion and exclusion criteria


**Inclusion criteria**	Diagnosis of SP (SCID-I)
	Liebowitz Social Anxiety Scale >30 (>60 for generalized subtype)
	Age 18 to 70 years
	SP must be primary diagnosis (most severe disorder according to ADIS-IV)
	SP patients with co-morbid disorders will be included, provided that SP is the primary diagnosis, thus ensuring a clinically representative sample as well as analyses of subgroups (for example, type of SP, patients with co-morbid depressive disorder)
**Exclusion criteria**	Psychotic disorder
	Prominent risk of self-harm
	Acute substance-related disorders
	Personality disorders except for avoidant
	Obsessive-compulsive or dependent personality disorder (SCID-II) organic mental disorder
	Severe medical conditions
	Concurrent psychotherapeutic or psychopharmacological treatment, with the following exception: specific psychopharmacological treatments (that is, tranquilizer, hypnotics, neuroleptics, phytopharmaceuticals, and beta-blockers prescribed as anxiolytics) should be terminated prior to therapy onset
	Anti-depressive medication represents no exclusion criterion as long it is reliably and stably consumed

### Interventions

**Figure 1:**
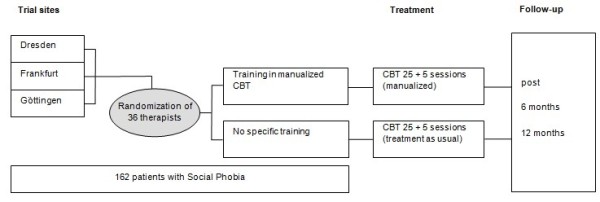
Intervention scheme/trial flow.

The experimental intervention comprises training in outpatient CBT for private practitioners, following the manual by Stangier, Clark, and Ehlers [22]. The manual follows the model of Clark and Wells [17] and prescribes interventions which are as follows: derivation an idiosyncratic version of the Clark and Wells model in cooperation with the patient; manipulation of self-focused attention and safety behaviors; application of video and audio feedback to modify distorted self-imagery; behavioral experiments in and out of the therapy room; attention shifts and interrogation of the social environment; identification and modification of anticipatory and post-event processing; identification of the patient’s dysfunctional assumptions and modification using cognitive restructuring. CBT treatment as usual will serve as the control condition. According to psychotherapy manuals (for example [23]), up to 25 + 5 sessions are scheduled. See Figure 1 for trial flow. Practitioners will receive two separate blocks of training based on Stangier *et al.*’s manual [22], each block with a mean duration of 12 h. Practitioners in both groups will also be offered regular group supervision (with a mean duration of 2 h).

### Assessment

**Figure 2:**
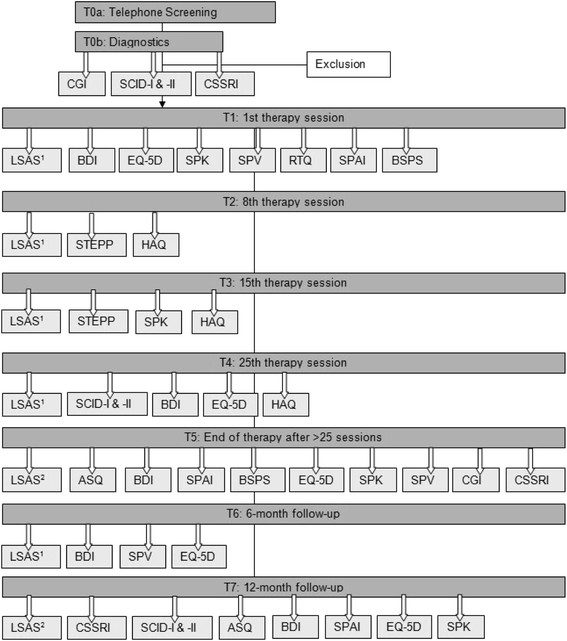
**Time points of assessment.** 1) LSAS self rating, 2) LSAS observer rating.

The time points of assessment are displayed in Figure 2.

Independent and trained SCID interviewers giving patient assessments are blind to the treatment condition. Assessments will be made at baseline, that is, prior to treatment onset (T1), at session 8 (T2), 15 (T3), and 25 (usually post-treatment, T4), at post-treatment (when treatment duration is longer than 25 sessions, T5). Every session is planned to have a 50-min duration. Follow-up assessments will take place 6 (T6) and 12 months (T7) after treatment; assessments at weeks 8 and 15 are performed to ensure intent-to-treat analyses. Primary endpoint: assessment after 25 sessions; secondary endpoint: post-treatment. Should treatment end at T4, measures for T5 will also be collected.

### Objectives and hypotheses

As a study combining the scientific benefits of randomized controlled trials (efficacy studies) and naturalistic studies (effectiveness research), its main objective is the analysis of the effects of additional training for private practitioners. Hence, the following question is investigated: does training in manualized treatment increase the effectiveness of psychotherapy for social phobia in natural settings?

We expect the effects of CBT on social phobia to be enhanced by training experienced therapists and by systematic transfer of innovative manualized treatments into practice. Thus, the specific hypotheses are as follows: (1) treatment effects achieved by private practitioners trained with the manualized procedures (Group 1) will be superior to those achieved by therapists who continue their standard treatment (Group 2); and (2) treatment duration (in terms of number of sessions between treatment onset and finalization) in Group 1 will be shorter.

### Outcomes

Endpoints and outcome measures were chosen in accordance with the main trial of the SOPHO-NET study [21]. Outcome measures include internationally used valid and highly relevant measures of SP. The primary outcome measure will be the Liebowitz Social Anxiety Scale (LSAS [24]). Remission from social phobia is defined as LSAS total scores ≤30 points. Secondary outcome measures include German versions of other standard scales for social anxiety (SPAI [25], SPK [26], SPV [27], BSPS [28]), depression (BDI [29]), and a scale for quality of life and social functioning (EQ-5D [30]). In addition we will assess emotional regulation (ASQ [31]), helping alliance (HAQ [32]), the subjective evaluation of sessions (STEPP [33]), as well as the reaction to treatment (RTQ [34,35]). During diagnostics, a rating on symptoms severity using the Clinical Global Impression scale (CGI [36]) is also given. The majority of these measures will also be used in a related trial of manualized short-term psychodynamic therapy allowing for high comparability between both trials [37]. Furthermore, patient sociodemographic data and costs of SP treatment are assessed using selected items of the German adaptation of the client sociodemographic and service receipt inventory (CSSRI; for example number of treatment sessions, days of work disability [38]). All instruments will be applied in both groups of this trial. Using established cut-off scores for LSAS (≤30 points [24]), the percentages of patients defined as remitted will be assessed and statistically compared between the group treated by specifically trained therapists and the group receiving standard CBT.

### Sample size calculation

The only study directly comparing performance of therapy delivered under RCT conditions and routine care refers to group CBT for social phobia [18] and showed a small but significant advantage for the RCT therapies. The difference between the Clark trials [17,18] and other controlled trials suggests a much higher difference in the to-be expected between-group effect sizes of our study but only under the improbable assumption that trained private practitioners will be comparably successful as the specialized therapists in the Clark trials. Sholomskas *et al.*[39], in a study directly testing the effects of additional training, but not in the field of SP, reported an average between group effect size of d = 0.69 when comparing therapists who were trained in a seminar plus supervision and therapists who used a manual without further training with regard to their performance in standardized role plays. However, this estimation does not directly refer to the expected effect sizes for the therapeutic benefit, which is the main outcome criterion of this trial. Given this inconsistent information, we choose to expect a more conservative effect size of d = 0.50 between Group 1 (trained therapists) and Group 2 (treatment-as-usual), for pragmatic reasons. We aimed at estimating the sample size needed for a dichotomous approach (remission rates according to the above defined criterion, LSAS <30). In order to assure comparability with the parallel trial for psychodynamic therapy [37] we introduced the same conservative assumptions: (1) the ICC was set to 15% which is high compared to similar studies [40]; and (2) for the primary analysis (ITT) drop-outs will be analyzed as patients with no remission. When assuming <50% remitters and an equal drop-out rate in both conditions, the absolute difference between remission rates will remain unchanged, but the relative difference will be much greater and will substantially enlarge the difference between treatments.

In contrast to Wiltink *et al.*’s study protocol [37], the average number of patients in a cluster was assumed to be five, as we choose to collaborate more continuously with the included therapists. Please note that this is the only assumption differing from those made in [37] resulting in a higher *a priori* sample size than in [37].

In order to detect a difference between Groups 1 and 2 at α = 0.05 (one-tailed; see Discussion) with a power of 0.80, *n* = 58 patients per group are required according to the formula of Campbell *et al.*[40,41] for cluster-randomization, given that five patients per therapist will be treated (for a more comprehensive account, see [42]):

$$N=2*{\left[\frac{\sigma (z_{\alpha }+z_{\beta })}{E}\right]}^{2}*\left[1+(m-1)\rho \right]=2*{\left[\frac{\left(z_{\alpha }+z_{\beta }\right)}{ES}\right]}^{2}*\left[1+(m-1)\rho \right]$$

(z_α_, z-value for probability of error type I alpha; z_β_, z-value for probability of error type II beta; σ, standard deviation of outcome; E, expected ES of outcome; m, mean observations for each cluster; ρ, Intracluster correlation coefficient (ICC); ES = E/σ, standardized effect size)

Given a conservative drop-out rate estimate of 25% (taking into account slightly elevated drop-outs in a practice study), a total of *n* = 162 patients are required to be allocated to the trial. Thus, *n* = 54 patients will have to be included in each center. According to data of the Stangier *et al.*[43] study on social phobia, in which patients were recruited in a comparable setting of German psychotherapeutic care, 61% of 250 patients meeting the inclusion criteria can expected to be finally included. We estimate that not more than about 250 patients will have to be assessed for eligibility.

### Randomization

Therapists will be randomly assigned either to a training group (manualized CBT), in which they will undergo an intensive training of the treatment manual for social phobia, or to a control group (routine care), in which the non-manualized CBT standard treatment is applied. Private practitioners will be block randomized. Practitioner randomization will be stratified for each trial center using the nQuerie Advisor® 6.01 software program [44]. As participants in a naturalistic study, patients are not randomized and have no knowledge on whether or not the therapist they have freely chosen has been trained in manualized CBT. Diagnostic procedures are performed by independent, blinded, and specially trained assessors for diagnosis and observer-rated outcome measures. All diagnosticians are trained by one of the co-authors (US), who was responsible for quality assurance of CBT in the SOPHO-NET study and has extensive experience in conducting RCT of SP [43,45]. There will be multimethod assessment of specific and non-specific therapy outcome including both patient and assessor perspective; control for drop-outs, intent-to-treat analyses. Generalizability of results will be established by above defined inclusion and exclusion criteria (Table 1) and by the multicenter approach.

### Statistical analysis

#### Primary analyses

The following hypothesis will be tested: H0: μ manualized ≤ μ standard *vs.* H1: μ manualized > μ standardwhere μ manualized and μ standard are the true remission probabilities in the manualized treatment group and the standard routine care group after the treatment period, respectively.

Remission rates will be compared between specifically trained and standard CBT group via logistic regression using covariates for treatment, sex of the therapist, and experience of the practitioner (as in [37]). For repeated measures, as suggested by Wood *et al.*[46], random effects models will be fitted of outcomes taking into account potential drop-outs (for further reference, see [42] and [47]). Hypothesis 1 of this trial will be tested by the group*time interaction (with dummy variables coding the time effects at time points T4 to T7 as compared to baseline measurement).

Further analyses will include: comparison of remission rates across treatment groups at time points T4 to T7 using the chi-squared test of independence; cross-sectional analyses at T4 to T7 comparing both patients and therapists in the three study centers; analyses of covariance of outcomes at T4 to T7 considering patient variables, taking into account potential differences between patients recruited by trained *vs.* those recruited by non-trained therapists; and multiple regression analyses to test whether diverse covariates including process variables predict different treatment outcomes. Regression analysis will be applied to each treatment group separately. Should the results be similar, the sample will be pooled.

A descriptive analysis will be made on drop-outs and their covariates. Imputation methods will be considered if necessary following an assessment of the missing value structure [46].

#### Secondary analyses

Random effects analysis of treatment outcomes as a function of time will also be employed to analyze associations with questionnaire data and observer ratings (SPAI, SPK, SPV, BSPS, BDI, EQ-5D, ASQ, HAQ, STEPP, reaction to treatment, CGI). Moreover, analyses to be performed will include the following: cross-sectional analyses as above; analyses of subjects’ and therapists’ conditions regarded as subgroup; SP treatment costs (CSSRI) [38] will also be considered as ‘process variables’; again, a one-sided level of significance at α = 0.05 will be chosen (see Discussion).

### Safety aspects

Trial progress, monitor data management, and patient safety will be reviewed by the control center for clinical studies (KKS), the Ethics Committee (EC) of the Technische Universitaet Dresden (Germany), and the Data Monitoring and Safety Committee (DMSC).

### Medical complications

Only psychological conditions according to the International Classification of Diseases F00-F99 (‘Mental and Behavioral Disorders’) will be recorded and classified as adverse events. Any deterioration that results in discontinuation of therapy (including suicidal risk and hospitalization) will be notified immediately by the private practitioner. Changes in psychological disorders will also be notified by the therapist and assessed by the diagnostician after session 25 of treatment, as well as 6 and 12 months post-treatment.

### Ethical issues

The trial has been approved by the responsible Ethics Committee of the Technische Universitaet Dresden (EK: 183062010) and the Data Safety Monitoring Committee (DSMC). Prior to assessment, patients will be informed about design and procedures, and will be required to give informed consent and may withdraw at any point without any disadvantage. Participating patients will be provided with a treatment consistent with good clinical practice according to the EMEA guidelines ( http://www.emea.eu.int/pdfs/human/ewp/363503en.pdf) for the diagnosis and treatment of SP. Suicidal tendencies will be examined regularly by the private practitioner. Should the need arise, outpatient services will be available and admission to cooperating clinics will also be possible. For further care, patients that cannot be included in the study will either be added to the practitioner’s general waitlist for further diagnosis and treatment or receive information on alternative potential healthcare practitioners upon request.

## Discussion

In the last decade, several authors have emphasized the large gap between therapeutic treatments validated at university institutions and the treatments applied in ‘everyday practice’ (for example [20,48,49]). These researchers argue for an increasingly efficient and effective dissemination of evidence-based treatments in light of empirical data indicating that, while such psychological treatments are being developed for a variety of psychopathological conditions (for an overview for depression see [50], for anxiety [51]), their application into routine clinical practice remains impaired. The factors to which the cause of this problem has been attributed to include a lack of knowledge about the effective transmission of CBT skills, the mechanism of action of CBT, and the minimum dose that patients require, in addition to the difficulties in measuring quality of therapy [52].

As a means to bridge this gap, the present study (SOPHO-PRAX) represents a first attempt to test the transferability of new CBT treatment options to the private clinical practice under controlled conditions. SOPHO-PRAX thus implies a continuation of the multicenter project evaluating the efficacy of cognitive behavioral therapy (CBT) and psychodynamic short-term therapy (STPP) (SOPHO-NET [21]). The novelty of the present CBT trial lies in the advantage of combining the elements of randomized controlled trials and naturalistic studies. It will directly inform about the incremental effects of procedures established in a controlled clinical trial into practice. Study results may be of great relevance to healthcare policy, health insurance companies, and the statutory boards of psychotherapists. They may serve to improve quality of treatment and shorten the timeframe between the development and widespread use of effective methods, thereby reducing health cost of expenditures [53]. The design of SOPHO-PRAX thus aims to further a faster and more widespread dissemination of effective treatments. As one study center is located in East Germany, study results will be of specific importance for deliverance of evidence-based psychotherapeutic interventions in this region and its unmet needs for psychotherapy.

The present study will also be the first to include systematic CBT training of private practitioners in the field of psychotherapy in the German healthcare system. It will help clarify whether clinical practitioners will generally regard the new CBT approaches as helpful for their work, and ascertain to what degree they will integrate the new methods into their clinical routine. As we specifically expected the new methods to be superior, it needs to be mentioned that we choose one-sided testing and an alpha of 0.05 as appropriate for a superiority trial [54]. There are two reasons for this decision. Firstly, in the field under investigation there is clear and robust evidence (not just expectation) that the manualized approach of CBT for social phobia according to Clark and Wells is the most efficacious among other CBT treatments (see [15]). It follows that the scientific hypothesis to be tested is one-sided. Secondly, as our study approach is that of a transfer or dissemination trial of an already established manualized procedure, only a clear advantage of this procedure over the reference intervention (non-manualized treatment) would have consequences for practice. Given these two conditions (that is, one-sidedness of scientific hypothesis and practice relevancy for superiority of the to-be implemented treatment only), we followed the recommendation of authors, such as Bland and Altmann [55] or Knottnerus and Bouter [56], and calculated the *a priori* sample size estimation based on one-sided testing. This approach warrants that the sample size is not larger than needed and the study is economic.

However, the clear methodological disadvantage of this decision has to be emphasized: Although it is not our scientific hypothesis, we can certainly not rule out ‘harm’, which in our case would mean superiority of the (non-manualized) reference treatment over the to-be implemented manualized one. Given the logic of the one-sided analysis, no statistical testing whether the standard procedure is superior to the manualized one can be made. Accordingly, a negative result in a superiority trial (like ours) would not prove that the investigated therapies are equivalent [54].

Nevertheless, the results of this study will not only inform about the degree to which the new methods lead to an improvement of treatment course and outcome, but also about whether the effects of routine psychotherapeutic treatment are comparable to those of the controlled, strictly manualized treatments of the SOPHO-NET study [21]. Finally, this study will allow an estimate of the reduction in treatment duration and costs made possible through implementation of treatment manuals into clinical practice. All these questions will be examined in a methodologically rigorous manner, as they seem highly relevant both for the domain of social phobia and the dissemination of psychosocial treatments in general.

### Trial status

Patient recruitment for the present trial is ongoing and expected to be completed by September 2013. Therapies are expected to be completed in 2014.

## Abbreviations

ANOVA: Analysis of variance; ASQ: Affective Style Questionnaire; ADIS-IV: Diagnostic interview; AE: Adverse event; BDI: Beck Depression Inventory; BMBF: Federal Ministry of Education and Research; BSPS: Brief Social Phobia Scale; CBT: Cognitive behavioral therapy; CRF: Case report form; CGI: Clinical Global Impression scale; CSSRI: Client Sociodemographic and Service Receipt Inventory; DMSC: Data Monitoring and Safety Committee; DSM-IV-TR: Diagnostic and Statistical Manual of Mental Disorders 4th edition, text revision; EC: Ethics Committee; EQ-5D: Quality of life questionnaire; ES: Effect size; HAQ: Helping Alliance Questionnaire; ICC: Intracluster correlation coefficient; ICD-10: International Classification of Diseases; ITT: Intention to treat; KKS : Control center for clinical studies; LOCF: Last observation carried forward; LSAS: Liebowitz Social Anxiety Scale; RCT: Randomized clinical trial; SAD: Social Anxiety Disorder; RTQ: Reaction to treatment questionnaire; SAD: Social anxiety disorder; SAE: Serious Adverse Event; SCID: Structured Clinical Interview for DSM Disorders; SOPHO-NET: Social Phobia Psychotherapy Research Network; SOPHO-PRAX: Study on the transfer of manualized CBT for social phobia into clinical practice; SP: Social Phobia; SPAI: Social Phobia and Anxiety Inventory; SPK: Social Cognitions Questionnaire; STPP: Short-Term Psychodynamic Psychotherapy; SPV: Social Behavior Questionnaire; STEPP: Session evaluation sheet; T1-T7: Time points of assessment.

## Competing interests

The authors declare that they have no competing interests. JH received speaking honoraries from Astra-Zeneca for a project unrelated to the present trial.

## Authors’ contributions

SC and JH completed the first draft of the manuscript. JW, EL, and US added significant content to the first draft and contributed to its critical revision. JH, EL, US, SC, and JW substantially contributed to the conception and the design of the study. All authors read and approved the final manuscript.
